# Regulation of BZR1 in fruit ripening revealed by iTRAQ proteomics analysis

**DOI:** 10.1038/srep33635

**Published:** 2016-09-29

**Authors:** Lihong Liu, Haoran Liu, Shuo Li, Xin Zhang, Min Zhang, Ning Zhu, Craig P. Dufresne, Sixue Chen, Qiaomei Wang

**Affiliations:** 1 Key Laboratory of Horticultural Plant Growth, Development and Quality improvement, Ministry of Agriculture, Department of Horticulture, Zhejiang University, Hangzhou 310058, P.R. China; 2 Department of Biology, Genetics Institute, University of Florida, Gainesville, FL 32610, USA; 3 Thermo Fisher Scientific, West Palm Beach, Florida 33407, USA; 4 Proteomics and Mass Spectrometry, Interdisciplinary Center for Biotechnology Research, University of Florida, Gainesville, FL 32610, USA; 5 Zhejiang Provincial Key Laboratory of Horticultural Plant Integrative Biology, Department of Horticulture, Zhejiang University, Hangzhou 310058, China

## Abstract

Fruit ripening is a complex and genetically programmed process. Brassinosteroids (BRs) play an essential role in plant growth and development, including fruit ripening. As a central component of BR signaling, the transcription factor BZR1 is involved in fruit development in tomato. However, the transcriptional network through which BZR1 regulates fruit ripening is mostly unknown. In this study, we use isobaric tags for relative and absolute quantitation (iTRAQ) labeling technology to explore important proteins regulated by BZR1 in two independent tomato transgenic lines over-expressing *BZR1-1D* at four ripening stages, identifying 411 differentially expressed proteins. These proteins were implicated in light reaction, plant hormone pathways and cell-wall-related metabolism, etc. The ‘light reaction’ metabolic pathway was identified as a markedly enhanced pathway by BZR1 during tomato fruit ripening. The protein level of a probable 2-oxoglutarate-dependent dioxygenase 2-ODD2, involved in gibberellin biosynthesis was significantly increased at all four developmental and ripening stages. The results reveal molecular links between BR signaling pathway and downstream components involved in multiple ripening-associated events during tomato fruit ripening, which will provide new insights into the molecular mechanisms underlying tomato ripening regulatory networks, and be potential in understanding BR-regulated fruit ripening.

The ripening of fruits, a highly-orchestrated and genetically programmed process involving a series of physiological and biochemical changes, brings about drastic alterations in color, texture, aroma, and sugar content of the fruit^1^. Tomato (*Solanum lycopersicum*) fruits are among the most important, widely grown and consumed fruit crops. As an established model of fleshy fruit, tomato fruit development and ripening process is well characterized and understood from a hormonal-regulatory perspective. During tomato fruit ripening, chlorophyll and organic acid contents are reduced, and the cell wall is extensively modified, while carotenoids, sugars, soluble solids and a number of volatiles are accumulated^2^. Tomato fruit ripening is regulated by both endogenous and exogenous signaling systems, including developmental genes, light, temperature, and phytohormones.

Owing to its crucial function in the ripening of climacteric fruits, the regulatory role of ethylene in tomato fruit ripening has been well characterized at molecular level^1^,^3^. Additionally, other phytohormones have also been documented to control tomato fruit ripening. Abscisic acid (ABA) are implicated in tomato fruit development and ripening by regulating carotenoid accumulation and building up for the storage capacity of plastids^4^,^5^. Gibberellin (GA) has long been recognized with its anti-senescence activity by delaying fruit degreening, which counteracts ethylene-induced chlorophyll degradation during fruit development. AUXIN RESPONSE FACTOR 4 (ARF4) was found to be involved in regulating fruit plastid accumulation by mediating the transcriptional up-regulation of *SlGLK*s in tomato fruit^6^,^7^. Our previous study showed that jasmonic acid (JA)-induced lycopene biosynthesis in tomato fruit might be independent of ethylene signal transduction^8^. However, more general aspects of phytohormone regulation besides ethylene during tomato fruit ripening have not been intensively studied.

Brassinosteroids (BRs), an essential steroidal phytohormone in plants, regulate a wide range of physiological and developmental progresses including cell elongation, photomorphogenesis, stress responses and disease resistance^9^,^10^. Extensive genetic, molecular and proteomic studies have identified components involved in BR signaling and revealed a signaling pathway that connects BR perception at the cell surface to activation of transcription factors in the nucleus^11^. BRs are perceived by the plasma membrane receptor kinase BRI1, and act through its well-defined signal transduction pathway to activate members of the BZR1/BES1 family transcription factors^9^,^10^,^12^. Dephosphorylated BZR1 and BES1 accumulate in the nucleus and regulate target genes expression^12^,^13^. A point mutation in *BZR1* results in a gain of function Arabidopsis mutant *bzr1-1D*, which leads to the accumulation of dephosphorylated BZR1 protein and activation of BR response^14^. Recent studies revealed that BZR1 regulated several biological processes, including light regulated cotyledon opening, cell division and differentiation^15^. In contrast, our understanding of BR functions remains incomplete especially in regulation of fruit development and ripening.

Our previous study demonstrated that *BZR1-1D* over-expression resulted in elevated carotenoid contents and enhanced quality attributes during tomato fruit ripening^16^. However, the mechanisms by which BZR1 contributes to the dynamic assembly and organization of the complex ripening process remain poorly understood. Proteome is a highly dynamic model for understanding the biological and biochemical processes in a cell or an organism. The isobaric tag for relative and absolute quantitation (iTRAQ™) is a technique capable of multiplexing up to eight different samples for relative quantification^17^. In recent years, iTRAQ-based proteomic has been performed on several fruits, including tomato^18^, pear^19^ and mandarin^20^. In current survey, we compared the proteome profile of two *BZR1-1D* transgenic lines *BZR1-1D#6* (*#6*) and *BZR1-1D#23* (*#23*) and their wild type (WT) at different stages using iTRAQ and identified proteins regulated by BZR1 in early developing and ripening fruits. The differentially expressed proteins are involved in photosynthesis, metabolism, cell structure, protein synthesis, energy balance, stress and defense. The proteome analysis identifies new candidates component for the regulatory network controlling tomato fruit development. Our results, therefore, provide new insights into the mechanisms by which BZR1 mediates BR responses and regulates downstream physiological changes in tomato.

## Results and Discussion

### Protein Identification and Quantification of *BZR1-1D#6* and *BZR1-1D#23* fruit

In the study, we utilized transgenic lines designated as *BZR1-1D#6* and *BZR1-1D#23*, in which *BZR1-1D* from Arabidopsis mutant *bzr1-1D* with increased accumulation of BZR1, was transformed into tomato. These two lines were chosen because they showed significantly enhanced quality attributes^16^. Fruit proteome profile of *BZR1-1D#6* and *BZR1-1D#23* at immature (IM), mature green (MG), breaker (B) and mature red (R) stage stages were explored using iTRAQ-based quantitative proteomics technology coupled with LC-MS/MS. The iTRAQ labeling including three independent biological replicates was shown in Supplementary Figure S1. Developmental and ripening parameters were described in material and methods.

**Figure 1:**
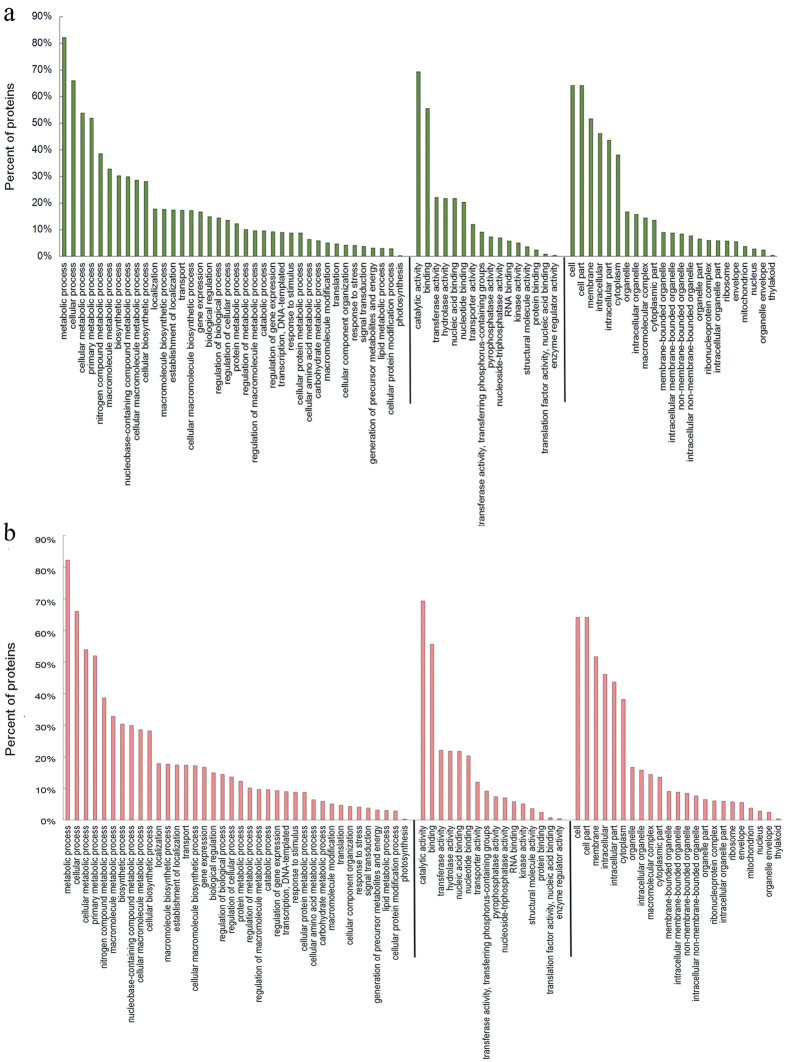
The GO terms in all the identified proteins. Biological process, Molecular function, Cellular component were mentioned. (**a**) The GO terms in all the identified proteins from fruit at IM and MG stage. (**b**) The GO terms in all the identified proteins from fruit at B and R stage. IM, immature green; MG, mature green; B, breaker; R, mature red.

A total of 2336 proteins were identified in three biological replicates. The detailed information of these proteins is shown in Supplementary Table S1 in the Supporting Information. The expression pattern of these proteins at different stages is shown in Supplementary Table S2 in the Supporting Information. Gene Ontology (GO) database was used to categorize all of the identified proteins, which cover a wide range of biological processes, cellular component, and molecular functions (Supplementary Table S1). The top two dominant terms were ‘cell’ and ‘cell part’ in cellular component, ‘catalytic activity’ and ‘binding’ in molecular function, and ‘metabolic process’ and ‘cellular process’ in biological process in both IM-MG sets (Fig. 1a) and B-R sets (Fig. 1b).

### Fruit proteome difference between *BZR1-1D* transgenetic lines and WT at different stages

**Figure 2:**
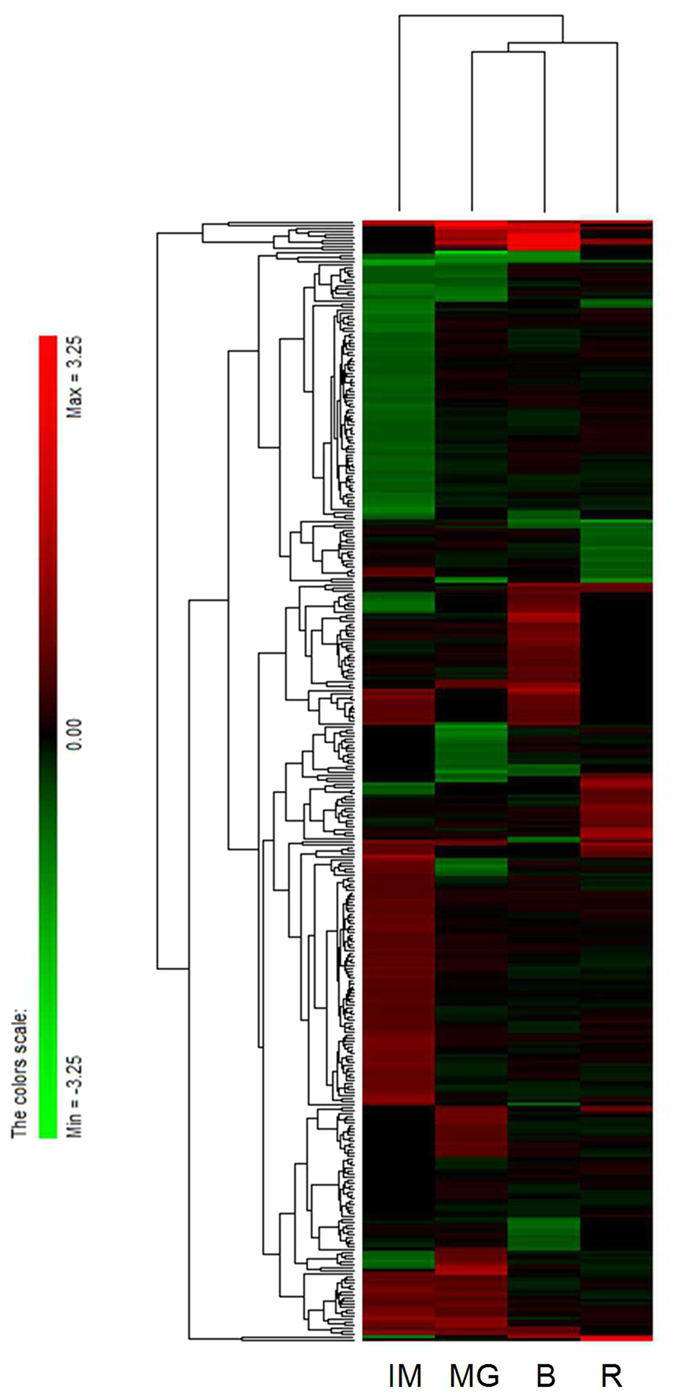
Hierarchical clustering of all differentially expressed proteins across different stages. The expression patterns of the proteins were hierarchically clustered based on the expression ratio as a log_2_ scale. Each row in the color heat map indicates a single protein. The green and red colors indicate down- and up-regulation, respectively, in the *BZR1-1D*s transgenic fruit relative to WT.

Expression ratios of the identified proteins between transgenic plants and WT at four stages were plotted in a Hierarchical clustering heat map on a log_2_ scale. As shown in Fig. 2, the regulation pattern of BZR1 at four developmental stages was different from each other. Moreover, BZR1-regulated proteins on the ripening stages of MG, B and R were similar, distinguishing between ripening stage and developmental stage (IM). These identified proteins were filtered to verify whether the changes in protein abundance are significant based on the cutoff values with a fold change < 0.667 or > 1.5, and p value < 0.05. A total of 97 proteins were finally identified as significantly altered in both *BZR1-1D#6* and *BZR1-1D#23* transgenic lines at one or more ripening stages. Among them, 50 proteins were up-regulated and 47 proteins were down-regulated in abundance in *BZR1-1D* transgenic lines compared to corresponding WT. Supplementary Table S3 shows these differentially expressed proteins along with the ratio of iTRAQ reporter ion intensities.

**Figure 3:**
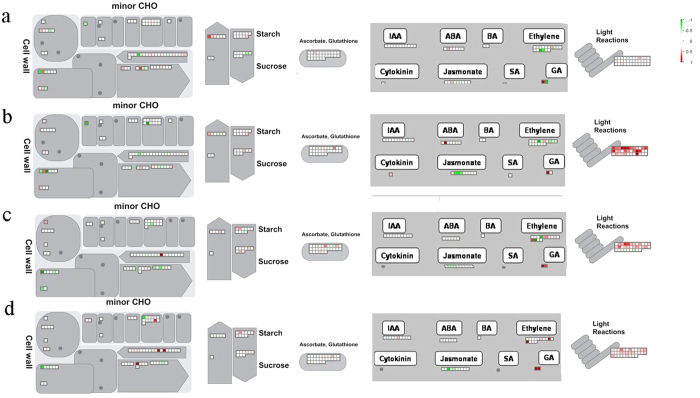
Regulatory and metabolic pathways of all differentially expressed proteins at different stages. Differentially expressed proteins in *BZR1-1D#23* transgenic fruit relative to WT at IM (**a**), MG (**b**), B (**c**) and R (**d**) stage were mapped in Cell wall, minor CHO, Starch, Sucrose, Ascorbate and Glutathione, Phytohormone, and Light Reactions. Individual tomato transcript matching the differentially expressed proteins were represented by small squares. Protein expression changes (log_2_ FC) were visualized on a color scale (red = induction, green = repression).

In our previous study, we found that *BZR1-1D#23* showed the most improved effect on tomato fruit quality trait^16^. Therefor, a MapMan overview of the differences in protein expression between *BZR1-1D#23* and WT is presented in Fig. 3. A full list of MapMan Bins is presented in Supplementary Table S4. The importance of several biochemical pathways, including cell wall, minor carbohydrate (CHO), starch, sucrose, sugars, ascorbate, glutathione, phytohormone and light reaction at MG (Fig. 3a), B (Fig. 3b), P (Fig. 3c), and R stage (Fig. 3d) has been demonstrated. Although the changes in protein expression were relatively small, it’s clear that some proteins were up-regulated, especially those involved in the light reactions at MG stage. Interestingly, our results demonstrate that BZR1 regulates light signaling pathways in tomato fruit, which are consistent with the actions of BZR1 on cell elongation and photomorphogenesis reported in Arabidopsis^21^. This result also validated the phenotype of dark green shoulders in transgenic fruits at B stage in our former study^16^. BR-deficient mutants grown in the dark not only display light-grown morphology but also express light-induced genes^22^,^23^. The molecular mechanisms underlying this regulation remains to be fully determined^24^. Sun *et al*.^25^ found that BZR1 could directly regulate the key components of light signaling pathways, including photoreceptors and downstream effectors. Closer inspection revealed that the expression of proteins related to cell wall were up-regulated, including endo-1,4-beta-xylanase (Solyc05g051260.2.1) at IM stage, pectin methylesterase (PME, Solyc07g064180.2.1) at IM and MG stages and GDP-l-fucose synthase (FX, Solyc07g006070.1.1) at R stage (Table 1). At the same time, some proteins down-regulated by BZR1 were also related to cell wall, including pectinesterase (PE1, Solyc07g064170.2.1) at MG stage, UDP-glucose 4-epimerase (UGE1, Solyc08g080570.2.1) at IM and MG stages, pectate lyase 1–27 (Solyc06g083580.2.1) at B stage, pectin methylesterase (PMEU1, Solyc03g123630.2.1) at all the four stages (Table 1).

Because plant hormones are known to play essential roles in fruit development and ripening, we explored the coverage of proteins associated with biosynthesis and signaling pathways connected to the major phytohormones. Sun *et al*.^25^ reported that BZR1 could directly regulate a number of genes involved in biosynthesis of GA, ethylene and JA. Then, BR could crosstalk with other signaling pathways to co-regulate the common transcriptional targets, as well as modulate the expression levels of key components in other signaling pathways to affect the sensitivity of plants to other signals and indirectly alters the expression of genes downstream of these pathways^26^. Interestingly, proteins associated with GA synthesis-degradation displayed altered expression in the transgenic lines of our present work. 2-oxoglutarate-dependent dioxygenase (2-ODD1, Solyc02g062460.2.1, implicated in GA biosynthesis) was repressed at IM stage and induced at B and R stage; whereas 2-oxoglutarate-dependent dioxygenase (2-ODD2, Solyc02g062500.2.1, implicated in GA biosynthesis) was significantly up-regulated at all the four stages. BR and GA are two major growth-promoting hormones that have similar effects on a wide range of developmental processes. Mutants deficient in either BR or GA show various degrees of dwarfism and reduced growth. Recent studies suggest that BR and GA crosstalk at multiple levels. Early studies found that BR induces the expression of GA biosynthetic genes, including *GA5*^27^, GA20ox-1, GA20ox-2, and GA20ox-5, in Arabidopsis^28^. Under low BR levels, BR induces GA biosynthesis and inhibits GA inactivation in rice^29^. Induction of GA production by BR to release DELLA repressive effects on BES1/BZR2 in a feed-forward mode was observed in Arabidopsis^30^. Our results support the conclusion that BR can affect GA biosynthesis.

In addition, increment in ethylene production by application of BR was found in tomato fruit^31^. BRs positively influence ET biosynthesis through regulation of 1-aminocyclopropane-1-carboxylate (ACC) synthetase (ACS) and ACC oxidase (ACO) activities^32^. In our study, we found ACO6 (Solyc02g036350.2.1) and its homolog (ACO3, Solyc09g089580.2.1) were repressed by BZR1-1D at IM and B, and MG stages, respectively. The interaction of BRs and JA plays crucial roles in plant development and stress responses. Ren *et al*.^33^ found BRs negatively, regulate JA-induced inhibition of root growth in Arabidopsis. Our proteomics data showed that lipoxygenase (LOXcevi34, Solyc01g099160.2.1; LoxB, Solyc01g099190.2.1; LoxA, Solyc08g014000.2.1) in BIN 17.7.1.2 (jasmonate.synthesis-degradation) were all down-regulated by BZR1-1D at MG stages, although we previously found that both BR and JA could promote lycopene accumulation during tomato fruit ripening^8^,^16^. The results demonstrate a complex regulation network that contains multiple layers of regulators and integrates multiple signaling pathways during tomato fruit ripening.

### Protein changes in *BZR1-1Ds* at IM stage

Among the 17 differentially expressed proteins, 9 proteins were up-regulated and 8 proteins were down-regulated in *BZR1-1D* transgenic fruit at IM stage (Supplementary Table S3). UDP glucose-4-epimerase (UGE1, Solyc08g080570.2.1) involved in cell wall synthesis was repressed by BZR1-1D, as well as protein (SEC14/SFH5, Solyc11g051160) of the SEC translocase system. SEC14 could be involved in Golgi vesicle transport of phosphoinositides in plant plastids. ACO, one of the key enzymes catalyzing the biosynthesis of ethylene was decreased in abundance in *BZR1-1D* fruit proteome. In *Arabidopsis*, *AtACO4* was a putative target of BZR1^25^. Thus, we hypothesized that BZR1 negatively regulated expression of ACO and etheylene production at early developmental stage of fruit ripening. 2-oxoglutarate-dependent dioxygenase (2-ODD, Solyc02g062460.2.1, Solyc02g062500.2.1) participates in synthesis of secondary metabolites of plants, including phytohormone ethylene, GA, and flavonoids^34^,^35^,^36^. Protein level of 2-ODD was increased while its homologue was decreased in *BZR1-1D* transgenic fruit. Differential regulation of 2-ODD and its homolog may be due to the fine control of BZR1 on homologous genes. In our analysis, γ-aminobutyrate transaminase (GABA-TP1, Solyc07g043310.2.1) displayed a reduced abundance in *BZR1-1D* transgenic fruit at IM stage. GABA is a four-carbon non-protein amino acid and has the ability to lowering blood pressure in humans^37^. Thus, the effects of GABA on human health have been the subject of a substantial amount of attention in food production^38^. GABA is one of the most abundant amino acids in tomato fruits^38^. GABA-TP1 converts the GABA to succinic semi-aldehyde and is involved in the GABA shunt pathway during fruit ripening. Prostaglandin E synthase 3/ripening regulated protein DDTFR8 (Solyc04g072160.2.1) involved in arachidonic acid metabolism was up-regulated by BZR1-1D. Fructose-bisphosphate aldolase (FBA2, Solyc02g062340.2.1), catalyzing a reversible reaction that splits fructose 1, 6-bisphosphate into the triose phosphates dihydroxyacetone phosphate (DHAP) and glyceraldehyde 3-phosphate (G3P), increased in *BZR1-1D* transgenic fruit at IM stage (Supplementary Table S3). It has been revealed that FBA2 played an essential role in glycogen metabolism. Increased activity of FBA in *Anabaena sp.* stimulated photosynthetic yield^39^,^40^. Previous ChIP-chip study in *Arabidopsis* indicated that *AtFBA5* was a BR-regulated BZR1 target, while *AtFBA1*, *AtFBA2*, *AtFBA4*, and *AtFBA6* were low stringency BZR1 targets^25^. BZR1 might target *FBA* to regulate tomato early development.

Fruit development was accompanied by oxidative process and defense response with accumulation of H_2_O_2_ and peroxided lipids in the membrane^41^. In the present study, 7 oxidative stress and defense-related proteins were differentially displayed in *BZR1-1D* transgenic fruit, including pathogenesis-related protein-like protein (STH-2, Solyc02g031950.2.1), peroxidase (LecAPX2, Solyc02g079510.2.1), glutathione S-transferase (GST, Solyc01g081270.2.1), aquaporin 1 (PIP2.6, Solyc11g069430.1.1), and programmed cell death protein 5-like (PCDP5, Solyc02g071150.2.1) as well as small heat shock protein (sHSP, Solyc08g078700.2.1, Solyc06g076520) of well-known chaperone function in fruit ripening and heat shock stress. Among them, STH-2, LecAPX2, PCDP5, and sHSP were up-regulated, while GST and aquaporin 1 was down-regulated. LecAPX2 is an isoform of ascorbate peroxidase, which is involved in scavenging intracellular H_2_O_2_ in plants. sHSP has been described for responding to a wide range of environmental stresses, including oxidative stress^42^. GST has the ability of detoxification of xenobiotics and has been proven to act as GSH-dependent peroxidases to limit oxidative damage^43^. Differential regulation of oxidative stress-related proteins indicated an activation role of BZR1 in stress responsive process during tomato fruit early development, but the underlying functional mechanism needs to be further investigated.

### Protein changes in *BZR1-1Ds* at MG stage

At MG stage, 59 proteins were differentially regulated, including 28 up-regulated and 31 down-regulated (Supplementary Table S3). Tomato fruit ripening is accompanied by changes in photosynthetic activity. Consistent with other proteomic studies^44^, a widespread up-regulation was observed at MG stage for photosynthesis-related proteins, including Chlorophyll a-b binding proteins (Lhcb2, Solyc07g047850.2.1; Lhcb13, Solyc12g011450; Lhcb6A, Solyc09g014520), the oxygen evolving complex (OEC) proteins (PsbP, Solyc07g044860; PsbO1, Solyc02g065400; PsbO2, Solyc02g090030), PsbP-like protein chloroplastic-like (Solyc03g114930.2.1) and photosystem I reaction center protein subunit (PsaD, Solyc06g054260.1.1; Solyc07g066150.1.1). It has been reported that developing tomato fruits had the ability to conduct photosynthesis and contribute up to 20% of the photosynthate for fruits, with the rest provided by leaves^45^,^46^. Our previous work revealed that the contents of soluble solids and soluble sugar in *BZR1-1D#23* transgenic fruit at R stage were higher than those in WT^16^, which may be attributed to the increased accumulations of photosynthetic proteins and consequent enhancement of photosynthetic activity at MG stage.

The levels of UDP-glucose 4-epimerase (UGE1, Solyc08g080570.2.1), pectin methylesterase (PMEU1, Solyc03g123630.2.1) and pectinesterase (PE1, Solyc07g064170.2.1), involved in the regulation of cell wall carbohydrate biosynthesis and degradation of pectic cell wall components by polygalacturonase in ripening tomato fruit, were repressed in *BZR1-1D*s. Cell wall remodeling protein pectin methylesterase (PME) has potential ability to strengthen the cell wall by facilitating calcium cross-linking between adjacent pectin molecules and thus cell-to-cell adhesion^47^. This idea was supported by studies of the tomato *PMEU1* gene, an isoform of PME. Gene silencing experiments showed that the loss of *PMEU1* function leads to an enhanced rate of softening during ripening^48^. In the present study, the accumulation of PMEU1 protein was decreased in *BZR1-1D* transgenic fruit, which suggested that BZR1 was involved in texture changes of tomato fruits at IM stage.

A series of plants storage proteins, including legumin 11S-globulin (Solyc03g005580.2.1, Solyc09g025210.2.1, Solyc09g072560.2.1), vicilin-like protein (Solyc09g065470.2.1, Solyc09g082340.2.1, Solyc11g072380.1.1) and oleosin (Solyc03g112440.1.1, Solyc12g010920.1.1), showed significant up-regulation levels in *BZR1-1D* transgenic fruit. Globulin was part of the flavor quality of tomato fruit and good source of dietary protein, for producing leucine, tyrosine, glutamic acid, and aspartic acid. Oleosin was a major constitute of plant oil body, which was rich in triglyceride and existed in the cytoplasm of seeds and fruits. In addition to the cytoplasm oil body, plastid oil body was also found in many plant chloroplasts, elaiosome and chromoplast^49^. Carotenoids could be dissolved in lipids and stored in chloroplasts. The accumulation of carotenoids in tomato fruits was promoted in *BZR1-1D* transgenic plants, which may be due to the improvement of storage capacity by oleosin.

Proteins involved in oxidative stress and defense response showed decreased levels, including peptide methionine sulfoxide reductase (MsrA, Solyc03g111720.2.1), glutathione S-transferase (GST, Solyc01g081270.2.1), dehydration-responsive family protein (Solyc01g091690.2.1), oxidoreductase 2OG-Fe(II) oxygenase family protein-like (Solyc01g104130.2.1), peroxidase (LecAPX2, Solyc05g052280.2.1) and class I heat shock proteins (Solyc06g076540.1.1, Solyc09g015020.1.1). MSRA could be induced by ethylene during fruit ripening and is involved in the repair of oxidized damaged proteins^50^. LecAPX2 was reported to participate in programmed cell death and stress responses^51^ and its transcript expression decreased as onset of fruit ripening^52^. Modulation of stress-related proteins suggested that BZR1 played an important role in keeping redox homeostasis. In addition, combined with the up-regulation of photosystem-related proteins, we may infer that BZR1 was a hub in balancing fruit growth and immunity, to allow more energy for growth.

### Protein changes nin *BZR1-1Ds* at B stage

At B stage, 11 differentially expressed proteins in fruit of *BZR1-1D*s were identified, 6 of them were up-regulated and 5 proteins were down-regulated (Supplementary Table S3). Consistent with results at MG stage, the most remarkable change at B stage was the high accumulation of probable fructose-bisphosphate aldolase (FBA2, Solyc02g062340.2.1), 2-ODD (Solyc02g062460.2.1, Solyc02g062500.2.1), chlorophyll a-b binding protein (Lhcb2, Solyc07g047850.2.1), subtilisin-like protease-like (Solyc08g079870.1.1) and lipid transfer protein (Solyc10g075070.1.1). The functions of subtilisin-like protein and lipid transfer protein in fruit ripening remain to be determined. Moreover, ACO6 (Solyc02g036350.2.1) remained down-regulated by BZR1-1D at B stage.

### Protein changes in *BZR1-1Ds* at R stage

At R stage, 10 differentially expressed proteins were identified in *BZR1-1D* transgenic fruit. In line with the expression pattern at previous developmental stage, non-specific lipid-transfer protein (nsLTP) and storage proteins such as legumin 11S-globulin and oleosin were up-regulated; cell wall remodeling protein pectin methylesterase and pectinesterase were down-regulated in *BZR1-1D* transgenic fruits. The 2-ODD up-regulated at IM, MG and B stages also showed an increased protein level at R stage in fruit of *BZR1-1D* transgenic tomato.

### Characterization of proteomic changes during stage conversion from IM to MG and B to R

**Figure 4:**
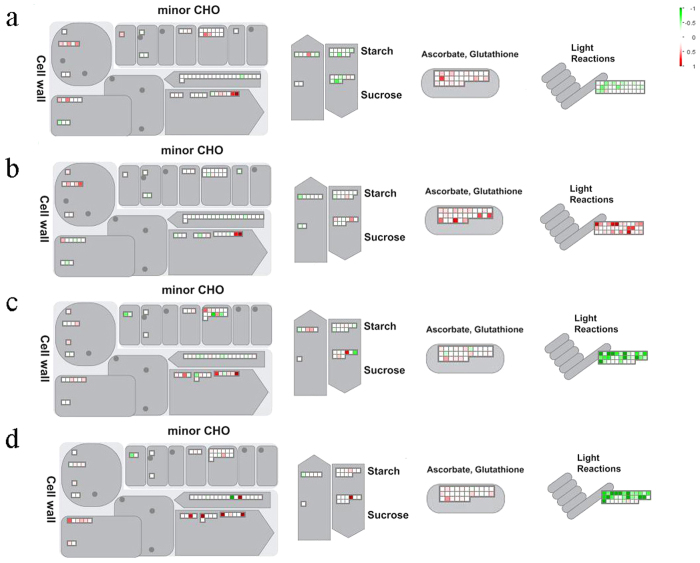
Regulatory and metabolic pathways of all differentially expressed proteins during stage transition. Differentially expressed proteins from IM to MG in WT (**a**) and *BZR1-1D#23* (**b**) fruit, and from B to R in WT (**c**) and *BZR1-1D#23* (**d**) fruit were mapped in Cell wall, minor CHO, Starch, Sucrose, Ascorbate and Glutathione, and Light Reactions. Individual tomato transcript matching the differentially expressed proteins were represented by small squares. Protein expression changes (log_2_ FC) were visualized on a color scale (red = repression, green = induction).

**Figure 5:**
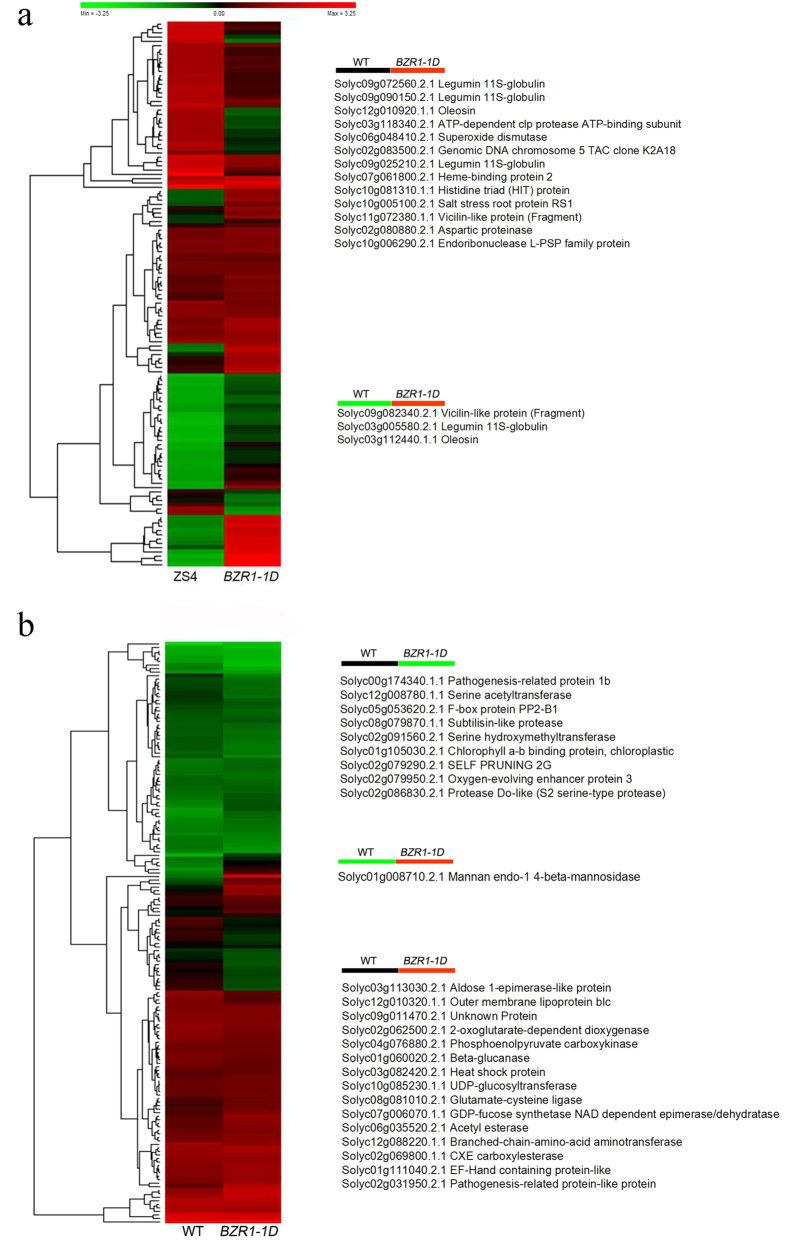
The change tendency of proteins in *BZR1-1D#23* and WT fruit during ripening stage converting from IM to MG (**a**) and B to R (**b**). The expression patterns of the proteins were hierarchically clustered based on the expression ratio as a log_2_ scale. Each row in the color heat map indicates a single protein. The green and red colors indicate down- and up-regulation, respectively.

A MapMan overview of the differences in protein expression between *BZR1-1D#23* and WT during stage conversion from IM to MG and B to R is presented in Fig. 4. The comparisons of fruit proteomic changes during conversions from IM to MG and B to R between *BZR1-1D* transgenic tomato (Fig. 4b, conversions from IM to MG; Fig. 4d, conversions from B to R) and WT (Fig. 4a, conversions from IM to MG; Fig. 4c, conversions from B to R) provided dynamic developmental information of fruit ripening. Proteins with the differentially displayed pattern in *BZR1-1D* transgenic fruit and WT during two conversions were clustered and shown in Fig. 5.

During stage conversion from IM to MG, the light reaction in WT was repressed, whereas that in *BZR1-1D* plants was enhanced (Fig. 4a,b), which is the most remarkable difference between *BZR1-1D*s and WT during the dynamic stage change (Table 1). Compared to the proteins showing different expression patterns between adjacent developmental stages in WT and BZR1-1D transgenic fruits, the proteins with a opposite accumulation were mainly enriched on the enhancement of storage proteins from IM to MG (legumin 11S-globulin, Solyc03g005580.2.1; vicilin-like protein, Solyc09g082340.2.1; oleosin, Solyc03g112440.1.1) and Mannan endo-1,4-beta-mannosidase (Solyc01g008710.2.1) from B to R (Fig. 5). The increase of storage related proteins in *BZR1-1D* transgenic plants occurred at MG, B and R stage, indicating a positive regulation by BZR1 in developmental tomato fruits. Tomato fruit ripening related enzyme endo-1,4-beta-mannosidase participates in the degradation of cell walls and locates in the cell wall of tomato fruits^53^. The findings may explain the approximate 1 week earlier ripening time of *BZR1-1D* transgenic fruit compared to WT^16^.

**Figure 6:**
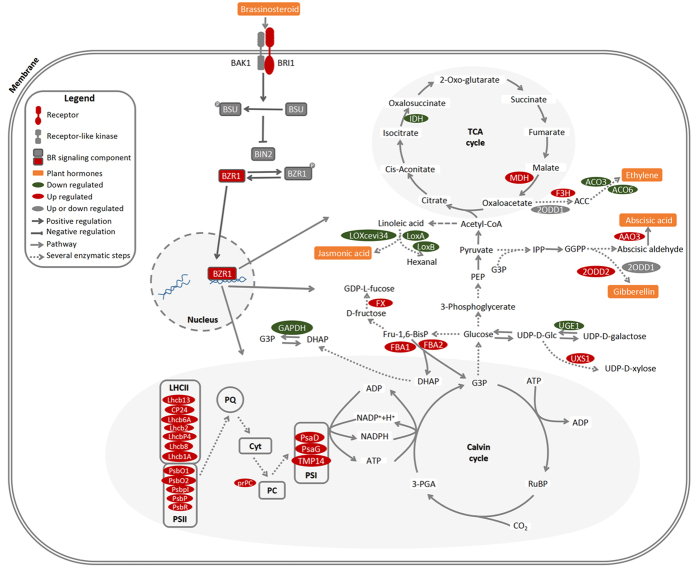
Schematic model of BZR1-regulated proteins in tomato fruit. The red ellipse represents proteins up regulated by *BZR1-1D* overexpresion in tomato fruits at one or more stages and showing no down regulation at any stage detected. The green ellipse represents proteins down regulated by *BZR1-1D* overexpresion in tomato fruits at one or more stages and showing no up regulation at any stage detected. The grey ellipse represents proteins showing different regulation pattern among different stages by *BZR1-1D* overexpresion in tomato fruits. AAO3, aba aldehyde oxidase; ACO3, 1-aminocyclopropane-1-carboxylate oxidase homolog; ACO6, 1-aminocyclopropane-1-carboxylate oxidase; CP24, chlorophyll (a-b binding protein cp24 chloroplastic-like; FBA1, fructose-bisphosphate aldolase 1; FBA2, fructose-bisphosphate aldolase 2; FX, GDP-l-fucose synthase(FX); F3H, flavanone 3 beta-hydroxylase; GAPDH, glyceraldehyde 3-phosphate dehydrogenase, solyc06g071920.2.1; IDH, isocitrate dehydrogenase, solyc01g005560.2.1; LhcbP4, chlorophyll a-b binding protein P4; Lhcb1A, chlorophyll a-b binding protein chloroplastic-like; Lhcb2, chlorophyll a b binding protein; Lhcb6A, chlorophyll a-b binding protein 6A, chloroplastic; Lhcb8, chlorophyll a-b binding protein 8, chloroplastic; Lhcb13, Chlorophyll a-b binding protein 13, chloroplastic; LoxA, lipoxygenase; LoxB, lipoxygenase; Loxcevi34, lipoxygenase; MDH, malate dehydrogenase, solyc09g01070; prPC, pre-plastocyanin; PsaD, photosystem I reaction center subunit2; PsaG, Photosystem I reaction center subunit V; PsbO1, 33kda precursor protein of oxygen-evolving complex; PsbO2, 33kda precursor protein of oxygen-evolving complex; PsbP, photosystem II subunit p-1; Psbpl, psbp-like protein chloroplastic-like; PsbR, photosystem II polypeptide; TMP14, thylakoid membrane phosphoprotein 14 kDa chloroplastic; UGE1, UDP-glucose 4-epimerase 1; UXS1, UDP-glucuronate decarboxylase protein 1; 2ODD1, 2-oxoglutarate-dependent dioxygenase; 2ODD2, 2-oxoglutarate-dependent dioxygenase.

Fruit ripening is a highly coordinated, genetically programmed, irreversible phenomenon that involves a complex series of physiological and biochemical events. Two *BZR1-1D* transgenic lines displayed higher carotenoid and fruit quality attributes consistently^16^. iTRAQ is a powerful quantitative tool in proteomic studies and has been suggested to be more sensitive than the Differential In Gel Electrophoresis (DIGE) technique^54^. In the present study, we performed a comprehensive iTRAQ-based quantitative proteome analysis of *BZR1-1D*s fruits at different developmental and ripening stages. The increased accumulation of photosynthesis proteins and extended photosynthetic reaction time may be the reason for improvement of fruit quality attributes in *BZR1-1D*s (Fig. 6). This proteomic analysis of *BZR1-1D*s tomato fruits at four different ripening stages may also provide insights into the metabolism and regulation of fruits ripening. The enhanced expression of proteins in GA biosynthesis and repressed expression of JA biosynthetic proteins by BZR1 confirm its central role in the BR signaling, which promotes or inhibits other signaling pathways to regulate plant growth and development (Fig. 6).

## Methods

### Plant materials and growth conditions

Transgenic plants *35S::BZR1-1D::CFP*#6 (*BZR1-1D*#6) and *35S::BZR1-1D::CFP*#23 (*BZR1-1D*#23) and their wild type (WT), Zhongshusihao (ZS4), were described in our previous work. These transgenic lines carry the mutational *BZR1* (*BZR1-1D*) gene driven by the cauliflower mosaic virus (CasMV) 35S promoter, which cause increased BZR1 accumulation^12^.

Plants were grown in greenhouse under 16 h-light/8 h-dark photoperiod at 22 °C (night) and 28 °C (day). Flowers were tagged at anthesis to follow fruit ages through development. Fruits of WT were harvested at immature green stage (IM, 15 days post-anthesis), mature green stage (MG, 43 days post-anthesis), breaker stage (B, 46 days post-anthesis) and R stage (red ripe, 56 days post-anthesis) of fruit ripening. Fruits of *BZR1-1D* were harvested a week earlier than WT at the corresponding ripening stages except IM stage. The number of fruits was limited to fewer than four per cluster. After collection, fruits were immediately frozen in liquid nitrogen and stored at −80 °C.

### Protein extraction and digestion

Proteins extraction from tomato fruits was performed as described with minor modifications^55^. Different stages of samples were finely powdered in liquid nitrogen and homogenized in protein extraction buffer (0.1 M Tris-HCl pH 8.8, 10 mM EDTA, 0.2 M DTT, 1.8 M sucrose), continued grinding in a fume hood, and then the extracts were agitated for 2 h at room temperature. After precipitating by ice cold 0.1 M ammonium acetate in methanol, the simply dried pellet was washed twice using precipitation buffer, twice with 80% acetone, and finally dissolved in 2D buffer (8 M Urea, 4% CHAPS, 40 mM Tris-base, 2 M Thiourea). Protein assays were performed wiyh EZQ Protein Quantitation Kit (Invitrogen, Carlsbad, CA, USA).

### iTRAQ Labeling and SCX Fractionation

50 μg protein from each sample was digested into peptides with trypsin (Promega, Madison, USA). Peptides were subsequently cleaned up with C18 desalting columns (The Nest Group Inc., USA) and then lyophilized to dryness. iTRAQ labeling was performed according to the manufacturer’s instructions for the iTRAQ reagents 8-plex kit (AB Sciex Inc., Foster City, California, USA). WT was labeled with iTRAQ tags 113 and 117; *BZR1-1D#6* was labeled with iTRAQ tags 115 and 119; *BZR1-1D*#*23* was labeled with iTRAQ tags 116 and 121 (Figure S1). For each sample of WT and transgenic lines, three independent biological replicates were performed.

After labeling, samples from the same set were combined together and lyophilized. The peptide mixtures were dissolved in strong cation exchange (SCX) solvent A (25% (v/v) acetonitrile, 10 mM ammonium formate and 0.1% (v/v) formic acid, pH 2.8). The samples were fractionated using Agilent HPLC 1260 with a polysulfoethyl A column (2.1 mm × 100 mm, 5 μL, 300 Å; PolyLC, Columbia, MD, USA) at a flow rate of 0.2 mL/min. After that, peptides were eluted with a linear gradient of 0–20% solvent B (25% (v/v) acetonitrile and 500 mM ammonium formate, pH 6.8) over 50 min, followed by ramping up to 100% solvent B in 5 min. 12 fractions were collected and lyophilized for LC−MS/MS analysis.

### LC−MS/MS Analysis

Each SCX fraction was resuspended in Solvent A (0.1% formic acid in 97% water, 3% acetonitrile). MS/MS analysis was carried out on a hybrid quadrupole Orbitrap (Q Exactive) mass spectrometer (Thermo Fisher Scientific, Bremen, Germany) with high energy collision dissociation (HCD). The MS system was interfaced with an automated Easy-nLC 1000 system (Thermo Fisher Scientific, Germerling, Germany). Each sample fraction was loaded on an Acclaim Pepmap 100 pre-column (20 cm × 75 μm; 3 μm-C18) and separated using a PepMap RSLC analytical column (250 cm × 75 μm; 2 μm-C18) with a flow rate at 350 nl/min. A linear gradient of solvent A (0.1% formic acid) to solvent B (0.1% formic acid, 99.9% acetonitrile) was run for 95 min, followed by a ramp to 98% B for 5 min. The instrument was run in a positive mode applying data dependent MS scan and MS/MS acquisition. Full scan MS spectra (400–2000 m/z) were acquired with resolution R = 70,000, 170000 intensity threshold, and fixed first mass = 105 m/z.

### Protein Identification and data analysis

Proteome Discoverer 1.4 with SEQUEST algorithm (Thermo Scientific Inc., Bremen, Germany) was used to search the original MS/MS protein data in the specified non-redundant databases (Uniprot, http://www.uniprot.org/uniprot/query=solanum+lycopersicum&sort=score, 39,529 entries; NCBI, http://www.ncbi.nlm.nih.gov/gquery/?term=solanum+lycopersicum, 38,078 entries; Phytozome, http://www.phytozome.net/tomato.php, 34,727 entries). Fixed modification of methylmethane thiosulfate-labeled cysteine, iTRAQ8plex (+304.205 Da (K) modification of amine groups in the N-terminus and lysine, and dynamic modifications included phosphorylation (+79.966 (S,T,Y)), oxidation (+15.995 Da (M)), carbamidomethyl (+57.021 Da (C)), iTRAQ8plex (+304.205 Da (K), and cysTMT6plex (+304.177 Da (C)) were considered. Proteome Discoverer nodes for spectrum grouper and spectrum selector were set using default parameters. Tolerances were set to a 10 ppm precursor mass tolerance and a 0.01 Da fragment mass tolerance. 1 maximum missed cleavage sites of trypsin digestion was allowed. Percolator was used for protein identification with parameters set with a strict target false discovery rate (FDR) at 0.01 and a relaxed target FDR at 0.05.

After peptide and protein identification, iTRAQ datasets were analyzed and fold changes were compared amongst respective reporter ions. Only proteins with at least 2 unique pepetides were considered. Fold-change < 0.667 or > 1.5) with P value less than 0.05 (with 95% confidence) is given to assess whether the protein is significantly differentially expressed. Significantly differentially expressed proteins detected in at least two biological repeats were considered to perform other analysises. The agriGO tool (http://bioinfo.cau.edu.cn/agriGO) was used to perform the Gene Ontology term enrichment analysis using SEA (Singular Enrichment Analysis) coupled with available background data of tomato^56^. A freely available program called PermutMatrix was used to carry out the clustering analysis^57^.

The tomato MapMan ontologies (http://www.gomapman.org/export/current/mapman/sly_SL2.40_ITAG2.3_2015-01-09_mapping.txt.tgz) were retrieved from the GOMapMan web resource^58^ and imported in the MapMan tool version 3.6.0^59^. Then, the tomato transcripts matching the identified proteins were mapped to bins for data visualization and pathway analysis.

## Additional Information

**How to cite this article**: Liu, L. *et al*. Regulation of BZR1 in fruit ripening revealed by iTRAQ proteomics analysis. *Sci. Rep.* **6**, 33635; doi: 10.1038/srep33635 (2016).

## Supplementary Material

Supplementary Information

Supplementary Dataset 1

Supplementary Dataset 2

Supplementary Dataset 3

## Figures and Tables

**Table 1:** List of 56 proteins differentially expressed in *BZR1-1D#23* transgenic tomato fruit at different stages^*a*^.

Accession Number^b^	Description	IM *#23*/WT	MG *#23*/WT	B *#23*/WT	R *#23*/WT	WT MG/IM	WT R/B	*#23* MG/IM	*#23* R/B
**Cell wall**									
Solyc05g051260.2.1	Endo-1 4-beta-xylanase	0.71	0.47	0.13	0.13	−0.17	−0.09	−0.48	−0.08
Solyc06g083580.2.1	pectate lyase 1-27	−0.14	−0.43	−0.63	0	0.4	−0.37	0.12	0.34
Solyc08g068150.2.1	burp domain-containing protein	−0.07	−0.1	−0.07	−0.45	0.76	0.35	0.8	−0.06
Solyc10g080210.1.1	Polygalacturonase (PG)	0.16	0.37	−0.22	−0.15	1.05	1.78	1.22	1.9
Solyc03g123630.2.1	pectin methylesterase (PMEU1)	−0.73	−0.6	−1.38	−0.85	0.42	0.12	0.46	0.68
Solyc07g064170.2.1	Pectinesterase (PE1)	−0.39	−1.29	−0.25	−0.44	0.61	0.36	−0.17	0.31
Solyc07g064180.2.1	pectin methylesterase (PME)	0.6	0.64	−0.09	0.01	−0.2	−0.17	−0.16	0.01
Solyc07g006070.1.1	GDP-l-fucose synthase (FX)	−0.31	−0.21	−0.08	1.5	−0.02	−0.07	0.06	1.55
Solyc08g080570.2.1	UDP-glucose 4-epimerase 1 (UGE1)	−0.82	−0.61	0.02	0.19	0.15	−0.11	0.17	0.06
Solyc10g085920.1.1	UDP-glucuronate decarboxylase protein 1 (UXS1)	0.14	0	2.84	1.09	−0.09	−0.3	−0.14	−1.98
**minor CHO**									
Solyc02g087770.2.1	aldose 1-epimerase-like	−0.08	−1.16	−0.61	0.02	0.63	−0.88	−0.39	−0.31
Solyc09g011240.2.1	aldo-keto reductase family 4 member c9-like	0.23	0.14	0.2	0.77	0.05	−0.58	0.03	0.15
**Starch, Sucrose**									
Solyc02g020980.2.1	4-alpha-glucanotransferase dpe2-like (DPE2)	−0.07	0.1	−0.36	−0.17	−0.58	0	−0.12	0.1
Solyc03g083090.2.1	soluble starch synthase	0	−0.41	0.17	−0.16	0.59	0.45	0.19	0.13
Solyc03g083910.2.1	vacuolar invertase	−0.55	−0.28	0.15	0.26	0.3	0.87	0.57	1.01
Solyc12g009300.1.1	sucrose synthase	−0.16	0.2	−0.34	0.16	−0.71	−0.73	−0.25	−0.21
**Ascorbate, Glutathione**									
Solyc09g007270.2.1	ascorbate peroxidase (APX)	0.18	−0.14	−0.04	0.05	0.72	0.23	0.32	0.32
Solyc11g018550.2.1	thylakoid-bound ascorbate peroxidase	0.08	0.46	0.45	0.6	−0.07	0	0.33	0.1
Solyc06g073460.2.1	glutathione peroxidase	−0.22	0.33	0.15	0.14	0.31	0.25	0.86	0.27
Solyc08g006720.2.1	glutathione peroxidase	−0.05	0.26	−0.07	−0.08	0.29	0.23	0.64	0.37
Solyc08g080940.2.1	glutathione peroxidase	−0.32	−0.05	0.25	−0.05	0.39	0.23	0.71	0.09
Solyc12g056230.1.1	probable glutathione peroxidase 8-like (GPX8L)	−0.26	−0.07	−0.22	−0.01	0.46	−0.1	0.68	0.16
**Phytohormone (ethylene)**									
Solyc02g036350.2.1	1-aminocyclopropane-1-carboxylate oxidase (ACO6)	−0.79	−0.23	−1.01	−0.22	0.22	−1.21	0.74	−0.29
Solyc02g062460.2.1	2-oxoglutarate-dependent dioxygenase (2-ODD1)	−0.91	−0.23	0.75	1.12	−0.34	−0.01	0.31	0.38
Solyc02g083860.2.1	flavanone 3 beta-hydroxylase (F3H)	0.32	0.12	0.37	0.9	0	−0.87	−0.12	−0.13
Solyc08g006770.2.1	probable 2-oxoglutarate fe -dependent dioxygenase-like	0.01	−0.41	−0.24	−0.36	0.45	0.66	0	0.44
Solyc09g089580.2.1	1-aminocyclopropane-1-carboxylate oxidase homolog (ACO3)	−0.07	−0.94	−0.18	−0.04	1.22	0.73	0.28	0.81
**Phytohormone (GA)**									
Solyc02g062460.2.1	2-oxoglutarate-dependent dioxygenase (2-ODD1)	−0.91	−0.23	0.75	1.12	−0.34	−0.01	0.31	0.38
Solyc02g062500.2.1	2-oxoglutarate-dependent dioxygenase (2-ODD2)	1.8	2.65	1.05	1.57	−0.29	−0.01	0.5	0.75
**Phytohormone (JA)**									
Solyc01g099160.2.1	lipoxygenase (LOXcevi34)	0.46	−0.81	−0.41	−0.88	1.11	0.52	−0.12	0.05
Solyc01g099190.2.1	lipoxygenase (LoxB)	−0.1	−0.78	−0.47	−0.35	1.05	1.03	0.38	1.09
Solyc08g014000.2.1	lipoxygenase (LoxA)	0.07	−0.66	−0.49	−0.1	0.62	−0.18	−0.01	0.26
**Phytohormone (ABA)**									
Solyc11g071620.1.1	aba aldehyde oxidase (AAO3)	0.51	1.15	N	N	−0.3	N	0.36	N
**Light Reaction**									
Solyc01g007500.2.1	photosystem II 47 kda protein (CP47)	0.04	0.26	0.29	0.39	−0.29	−0.59	−0.09	−0.57
Solyc01g105030.2.1	chlorophyll a-b binding protein cp24 chloroplastic-like (CP24)	−0.02	0.62	0.48	0.28	−0.2	−0.38	0.53	−0.64
Solyc02g065400.2.1	33kda precursor protein of oxygen-evolving complex (PsbO1)	0.02	0.6	0.17	0.36	−0.02	−0.76	0.5	−0.66
Solyc02g069460.2.1	photosystem I reaction center subunit chloroplastic-like	0.01	0.3	0.42	0.31	−0.48	−1.12	−0.09	−1.26
Solyc02g090030.2.1	33kda precursor protein of oxygen-evolving complex (PsbO2)	0.34	0.8	0.4	0.47	0	−0.87	0.46	−0.72
Solyc03g114930.2.1	psbp-like protein chloroplastic-like (Psbp1)	0.17	0.67	0.41	0.39	−0.24	−0.26	0.28	−0.27
Solyc04g082010.1.1	plastocyanin precursor (prPC)	0.24	1.11	0.25	0.37	0.11	−0.58	0.93	−0.37
Solyc06g054260.1.1	photosystem i reaction center subunit chloroplastic-like (PsaD)	0.15	0.64	0.41	0.41	−0.22	−1.12	0.3	−1.27
Solyc06g060340.2.1	photosystem II 22 kda chloroplastic-like (PsbS)	−0.02	0.51	0.44	0.39	−0.61	−0.06	0.07	−0.01
Solyc06g063370.2.1	chlorophyll a-b binding protein chloroplastic-like (Lhcb1A)	−0.06	0.63	0.44	0.23	−0.43	−0.42	0.34	−0.64
Solyc07g044860.2.1	photosystem II subunit p-1 (PsbP)	0.07	0.73	0.31	0.41	−0.08	−0.44	0.57	−0.41
Solyc07g047850.2.1	chlorophyll a b binding protein (Lhcb2)	0.13	0.97	0.78	0.61	−0.37	−1.01	0.52	−1.18
Solyc07g054290.1.1	thylakoid lumenal protein chloroplastic-like	0.19	−0.2	0.1	0.26	0.18	−0.75	−0.13	−0.69
Solyc07g066150.1.1	Photosystem I reaction center subunit V (PsaG)	0.15	0.97	N	N	−0.12	N	0.71	N
Solyc07g066310.2.1	photosystem II 10 kda chloroplastic (PsbR)	−0.14	0.58	0.4	0.49	−0.34	−1.16	0.32	−1.18
Solyc03g115900.2.1	Chlorophyll a-b binding protein P4 (LhcbP4)	−0.28	1.12	N	N	−0.46	N	0.87	N
Solyc09g014520.2.1	chlorophyll a-b binding protein chloroplastic-like (Lhcb6A)	0.02	0.6	0.47	0.25	−0.57	−1.03	0.06	−1.17
Solyc09g055950.1.1	Photosystem II D2 protein	0.2	0.48	0.67	0.21	−0.45	−0.72	−0.09	−1.09
Solyc10g005050.2.1	thylakoid membrane phosphoprotein 14 chloroplastic-like (TMP14)	−0.12	0.77	0.53	0.42	−0.14	−0.43	0.73	−0.53
Solyc10g007690.2.1	chlorophyll a-b binding protein chloroplastic-like (Lhcb8)	−0.08	0.84	0.71	0.33	0.06	−0.75	0.97	−1.23
Solyc10g017940.1.1	Photosystem II CP43 chlorophyll apoprotein (CP43)	−0.04	0.43	0.41	0.22	−0.64	−0.63	−0.03	−0.88
Solyc12g011450.1.1	Chlorophyll a-b binding protein 13, chloroplastic (Lhcb13)	−0.38	0.75	0.2	0.29	−0.21	−1.2	0.95	−0.88
Solyc12g033040.1.1	photosystem I p700 apoprotein partial	0.09	0.49	0.51	0.2	−0.53	−0.65	−0.09	−0.98

^a^Protein expression changes (log_2_ Fold Change) were shown in the table. ^b^Accession number and description from the International Tomato Annotation Group release version 2.3. N: not detected.
