# Supplementary material for: Regulation of BZR1 in fruit ripening revealed by iTRAQ proteomics analysis
Source: Sci Rep. 2016 Sep 29;6:33635. doi: 10.1038/srep33635 (PMC5041101; doi:10.1038/srep33635)
Supplement: Supplementary Information [file srep33635-s1.pdf]

Supplementary information

Regulation of BZR1 in fruit ripening revealed by iTRAQ proteomics analysis

Lihong Liu[1,4], Haoran Liu[1], Shuo Li[1], Xin Zhang[1], Min Zhang[1], Ning Zhu[4], Craig P. Dufresne[5], Sixue Chen[3, 4],Qiaomei Wang[1,2,*]

[1] Key Laboratory of Horticultural Plant Growth, Development and Quality improvement, Ministry of Agriculture, Department of Horticulture, Zhejiang University, Hangzhou 310058, P.R. China

[2] Zhejiang Provincial Key Laboratory of Horticultural Plant Integrative Biology, Department of Horticulture, Zhejiang University, Hangzhou 310058, China

[3] Proteomics and Mass Spectrometry, Interdisciplinary Center for Biotechnology Research, University of Florida, Gainesville, FL 32610, USA

[4] Department of Biology, Genetics Institute, University of Florida, Gainesville, FL 32610, USA

[5] Thermo Fisher Scientific, West Palm Beach, Florida 33407, USA

* *Corresponding author*

Corresponding author:

Qiaomei Wang

Professor

Key Laboratory of Horticultural Plant Growth, Development and Quality improvement, Ministry of Agriculture, Department of Horticulture, Zhejiang University, Hangzhou 310058, China.

Tel: 86 571 88982278

Fax: 86 571 88766022

Email: qmwang@zju.edu.cn

Supplementary Information Description

Supplementary Figures

Supplementary Figure S1. Label by iTRAQ reagent. iTRAQ labeling was performed according to the manufacturer's instructions for the iTRAQ reagents 8-plex kit (AB Sciex Inc., Foster City, California, USA). WT was labeled with tags 113 and 117; *BZR1-1D#6* was labeled with tags 115 and 119; *BZR1-1D#23* was labeled with tags 116 and 121 (Figure S1). For each sample of WT and transgenic lines, three independent biological replicates were performed.

Supplementary Tables

Supplementary Table S1-S4 are provided as separate excel sheets

Supplementary Table S1. Complete list of all 2336 proteins identified.

Supplementary Table S2. Expression pattern of proteins at different stages.

Supplementary Table S3. Differentially expressed proteins in both *BZR1-1D#6* and *BZR1-1D#23.*

Supplementary Table S4. Functional categories of proteins differentially expressed in *BZR1-1D#23.*

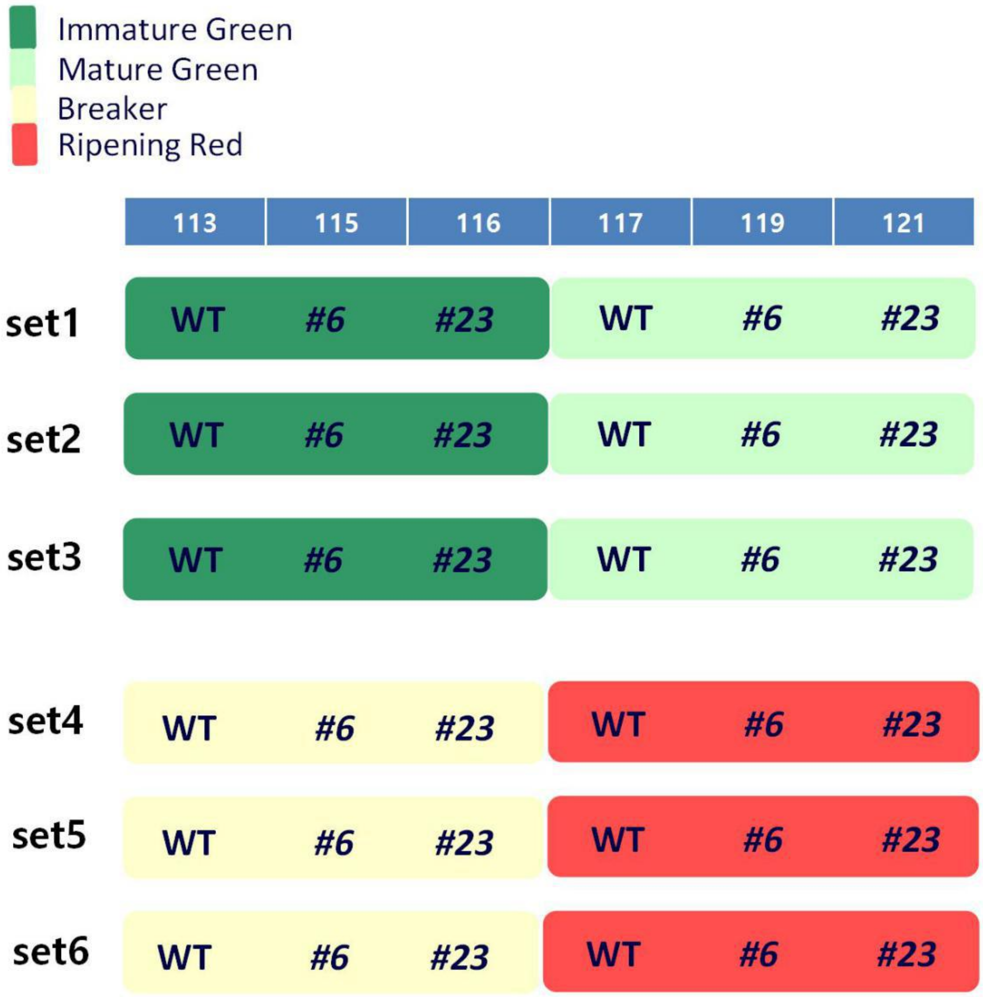

Supplementary Figure S1. Label by iTRAQ reagent. iTRAQ labeling was performed according to the manufacturer's instructions for the iTRAQ reagents 8-plex kit (AB Sciex Inc., Foster City, California, USA). WT was labeled with tags 113 and 117; *BZR1-1D#6* was labeled with tags 115 and 119; *BZR1-1D#23* was labeled with tags 116 and 121 (Figure S1). For each sample of WT and transgenic lines, three independent biological replicates were performed.
